# Expression of p16 and HPV E4 on biopsy samples and methylation of FAM19A4 and miR124‐2 on cervical cytology samples in the classification of cervical squamous intraepithelial lesions

**DOI:** 10.1002/cam4.2855

**Published:** 2020-02-05

**Authors:** Annemiek Leeman, David Jenkins, Marta del Pino, Jaume Ordi, Aureli Torné, John Doorbar, Chris J. L. M. Meijer, Folkert J. van Kemenade, Wim G. V. Quint

**Affiliations:** ^1^ DDL Diagnostic Laboratory Rijswijk The Netherlands; ^2^ Institute of Gynecology, Obstetrics and Neonatology Hospital Clínic—Institut d´Investigacions Biomèdiques August Pi I Sunyer (IDIBAPS), Faculty of Medicine‐University of Barcelona Barcelona Spain; ^3^ Department of Pathology ISGlobal Hospital Clinic of Barcelona Universitat de Barcelona Barcelona Spain; ^4^ Department of Pathology University of Cambridge Cambridge United Kingdom; ^5^ Amsterdam Medical Center Department of Pathology VU University Medical Centre Amsterdam The Netherlands; ^6^ Department of Pathology Erasmus University Medical Center Rotterdam The Netherlands

**Keywords:** cervical cancer, hrHPV, immunohistochemistry, LEEP, methylation

## Abstract

The decision to treat a cervical squamous intraepithelial lesion (SIL) by loop electrosurgical excision procedure (LEEP) relies heavily on a colposcopy‐directed biopsy showing high‐grade (H)SIL. Diagnosis is often supported by p16, an immunohistochemical (IHC) biomarker of high‐risk (hr)HPV E7 gene activity. Additional potential markers include methylation of tumor suppressor genes FAM19A4/miR124‐2 in cervical cytology for advanced transforming HSIL and the IHC marker HPV E4 for productive, potentially regressing lesions. In 318 women referred for colposcopy, we investigated the relationship between staining patterns of p16 and E4 IHC in the worst biopsy, and the relation of these to FAM19A4/miR124‐2 methylation status in cytology. E4‐positive staining decreased with increasing SIL/CIN grade from 41% in LSIL to 3% in HSIL/CIN3. E4 positivity increased with grade of p16 when p16 expression was limited to the lower two third of the epithelium (r = 0.378), but fell with expression over. Loss of E4 expression in the worst lesion was associated with the methylation of FAM19A4/miR124‐2. We also examined whether these biomarkers can predict the histological outcome of the LEEP biopsy in a subgroup of 119 who underwent LEEP treatment. About 85% of women with ≥lower two third p16 staining/E4‐negative HSIL biopsies and 65% with limited p16 staining/E4‐positive HSIL biopsies had ≥HSIL in the LEEP specimen (*P* = .025). p16 expression in a biopsy is related both to viral production and transformation, while decreased E4 expression relates to methylation, indicating advanced HSIL. p16 expression in ≥2/3 of the epithelium and absent E4 indicate likely HSIL on a subsequent LEEP specimen.

## Graphical abstract

We found an inverse relation between HPV E4 expression on biopsy and hypermethylation of FAM19A4/miR124‐2 on cytology sample. p16 and hypermethylation increased with the severity of the lesion and may be used to identify premalignant transformation. The use of p16/E4 immunostaining and methylation could offer valuable information beyond current grading practice.

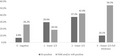

## 1 INTRODUCTION

In cervical cancer (CC) prevention, women with an abnormal screening result are subjected to colposcopy to detect of high‐grade squamous intraepithelial lesions/cervical intraepithelial neoplasia grades 2 and 3 (HSIL/CIN2‐3), or CC. Since histological diagnosis of HSIL/CIN2‐3 is the basis for surgical treatment, accuracy of diagnosis is key. However, hematoxylin and eosin stain (H&E) diagnosis is subjective and substantial interobserver and intra‐observer variability has been reported.^1^ Previous reports have shown that HSIL/CIN3 is more reproducible, but important variation exists in the diagnosis of HSIL/CIN2. This diagnosis includes both productive lesions that might regress and advanced transforming lesions that require treatment and can result in overtreatment.^2^, ^3^, ^4^

The use of biomarkers, whose results are unambiguous and reproducible, may provide a solution. Immunohistochemical (IHC) staining with p16, single or in combination with the proliferation marker Ki‐67, has been identified as a valuable marker in diagnosis of ≥HSIL/CIN2.^5^ p16 is a surrogate marker of the cell cycle deregulation by the high‐risk human papillomavirus (hrHPV) E7 gene. The HPV‐encoded marker panE4 is a novel marker for initiation of the viral productive phase and hence, completion of the papillomavirus life cycle.^6^ It is expressed in productive HPV infection in differentiated, mature epithelial cells.^7^, ^8^, ^9^ HSIL/CIN3 is almost always negative for E4, while HSIL/CIN2 and LSIL/CIN1 can be either E4‐positive or negative.^7^, ^9^ Current SIL/CIN classifications do not discriminate between different biomarker patterns corresponding to productive or transforming infection. Grading patterns of expression of biomarkers such as E4 and p16 might play an important role in predicting progression of a lesion, deciding treatment and might reduce the overtreatment of productive lesions that can regress.

Hypermethylation of the CpG islands in promotor regions of several tumor suppressor genes is extremely high in CC 10 and has been linked to the duration of hrHPV infection and the severity of the underlying neoplastic lesion.^11^ The combination of FAM19A4 and miR124‐2 shows particularly high levels of positivity in women with CC and high‐grade lesions with a longer duration of a preceding hrHPV infection.^12^ Lesions with a cancer‐like methylation pattern have been defined as “advanced” SIL/CIN lesions. The relation between hypermethylation detected on cytology sample, and p16 and E4 expression on biopsy has not previously been studied in a population undergoing routine colposcopy for an abnormal screening result.

This study aims to describe the relationships between the immunohistochemical expression patterns of markers p16 and HPV E4 in hrHPV‐positive colposcopy‐directed cervical biopsy, and also the relationship of these to methylation markers FAM19A4/miR124‐2 in cervical cytology samples of women referred to a colposcopy due to an abnormal screening test. In addition, we studied if biomarkers p16/E4 on biopsy and methylation on cytology can predict the histological outcome on loop electrosurgical excision procedure (LEEP) in a subgroup of 119 who underwent LEEP treatment.

## 2 METHODS

### 2.1 Population

The EVAH is a follow‐up study of women referred to the colposcopy clinic of Hospital Clínic, Barcelona, Spain because of abnormal cytology (≥ASC‐US).^13^ At colposcopy, women had a physician‐taken cervical cytology sample and 1‐4 colposcopy‐directed biopsies. Women were treated by LEEP when a HSIL/CIN2‐3 diagnosis was found on biopsy, when underlying HSIL/CIN2‐3 was expected on colposcopic impression, or when there was persisting HSIL cytology. Women were invited for a physician‐taken cytology sample and a colposcopy with biopsies when indicated every 6 months for 2 years. At the exit visit, a biopsy was taken in all women to collect a histological endpoint. All women enrolled before April 2014 were eligible for the present study. The study was approved by the medical ethical board of Hospital Clínic and signed informed consent was obtained from all women. This study is registered in the Dutch Trial register (NTR3464).

### 2.2 Collection of cervical cytology samples, hrHPV, and methylation testing

Physician‐taken cervical cytology samples were collected using a Cervex‐Brush (Rovers Medical Devices BV) and were rinsed in 20 mL of Thinprep medium (Hologic), before colposcopy. Samples were stored at room temperature for up to 6 months before DNA was isolated for hrHPV testing. An input volume of 250 μL was used to obtain 100 μL of eluate with the QIAamp MinElute Virus Spin kit (QIAgen Inc). hrHPV detection was performed using the GP5+/6+‐PCR‐EIA (Labo Bio‐medical Products). The EIA‐positive GP5+/6+ amplimers were genotyped using a strip‐based test by the Genotyping kit HPV GP (Labo Bio‐medical Products), genotyping hrHPV types 16, 18, 31, 33, 35, 39, 45, 51, 52, 56, 58, 59, 66, and 68.

Residual material after HPV testing and cytology was concentrated and sent to DDL Diagnostic Laboratory for methylation testing.^14^ DNA was isolated using the MagNA Pure 96. The level of amplifiable human DNA was assessed using a qPCR of the in‐house reference gene RNaseP with the phocine herpesvirus as an internal control for the absence of PCR‐inhibition.^15^ All samples with a DNA concentration of 2.2 ng/µL or higher were subjected to bisulfite conversion using the EZ DNA Methylation kit (Zymo Research). Samples with a DNA concentration below the threshold allowing reliable methylation testing in a physician‐taken sample were excluded. Up to 250 ng/45 µL of DNA was used, with 3 µL carrier RNA if samples contained less than 100 ng/45 µL. A standardized multiplex qMSP, targeting for the promotor regions of FAM19A4 and miR124 (QIAsure Methylation Test, Qiagen), was performed on the bisulfite‐converted DNA. The bisulfite‐converted human reference gene beta‐actin was included in the multiplex to determine the total amount of converted human DNA. Samples were scored methylation‐positive when at least one of the markers had a target gene/beta‐actin ratio above the threshold according to the kit insert. Results of marker FAM19A4 were also studied individually.

### 2.3 Sectioning and HPV testing of histology

Histologic samples were fixed in 10% neutral buffered formalin and embedded in paraffin following routine procedures. Four‐micrometre‐sections were obtained for H&E and p16 staining and these were assessed by an expert local pathologist following morphologic criteria supported by p16 staining according to the LAST recommendations.^5^ This diagnosis was used to decide treatment. Diagnoses on the LEEP specimens were made following the same procedure.

Biopsies were recut at DDL Diagnostic Laboratory, Rijswijk, The Netherlands according to the sandwich methods, producing one 4‐µm‐thick H&E before slide, two tubes with three 8‐µm‐thick sections for HPV testing, three 4‐µm‐thick slides for immunohistochemistry, a 4‐µm‐thick H&E after slide, and up to three 4‐µm‐thick back‐up slides. Diagnoses based on H&E and IHC recut slides were considered as the definitive diagnosis. DNA for HPV detection and genotyping was isolated using proteinase K isolation and SPF10‐PCR‐DEIA‐LiPA25 (version 1, Labo Biomedical Products BV). Women for whom the worst diagnosis on the local histology slide was more than one SIL/CIN grade different from the recut histology slides were excluded. This was done as the study histology could not be considered a reliable indicator of the lesion on which treatment was based. Those whose biopsy was hrHPV‐negative by SPF10‐PCR‐DEIA‐LiPA25v1 were also excluded.

### 2.4 p16 and E4 immunohistochemistry on biopsies

The biopsy with the worst diagnosis was then stained for p16, using p16^INK4a^ antibody (Roche, CINtec®, Clone E6H4™) and panHPVE4 mAb (LBP, Clone FH1.1), respectively, after heat‐induced epitope retrieval with citrate buffer. Reactivity was visualized using the EnVision Detection System.

Two expert pathologists, blinded to the local histological diagnosis, were asked to score the worst lesion for E4 and p16. Biomarker p16 was scored negative (0) when no staining or patchy staining was observed, diffuse staining restricted to the lower third of the epithelium was scored as 1, staining restricted to the lower two third of the epithelium was scored as 2 and staining above the lower third of the epithelium, including full thickness staining, was scored as 3. E4 staining was scored negative (0), focal when limited positive staining was seen in the superficial layer of the epithelium (1) or extensive when there was widespread‐positive staining in the superficial layer of the epithelium and or below (2). This grading system has been previously validated as reproducible.^9^, ^16^ p16 score ≥1 and E4 score ≥1 were considered positive scores for these markers.

To investigate further the relation between the expression of p16 and E4 and outcome we combined scores of p16 and E4, and used this to predict the presence of ≥HSIL/CIN2 and ≥HSIL/CIN3 on LEEP treatment. Lesions with p16 expression restricted to the lower third of the epithelium, or less, and all lesions with E4 positivity regardless of p16 expression were classified as combined IHC negative. In line with the LAST recommendations, all biopsies expressing p16 above the lower third of the epithelium,^5^ and no E4 were considered combined IHC positive.

### 2.5 Statistical analysis

Results were analyzed using IBM SPSS version 22.0 for Windows. The level of statistical significance was set at *P* ≤ .05 for all tests. Agreement between the diagnosis on the locally produced slide and the consensus diagnosis on the final selection of samples was calculated using Cohen's kappa. Correlation coefficients for HPV E4 positivity and methylation positivity were calculated using Spearman's correlation test and differences in positivity percentages were calculated using the Chi‐squared test. The authors confirm that the data supporting the findings of this study are available within the article.

## 3 RESULTS

A total of 538 women had a smear which was eligible for methylation testing and interpretation of the result.^14^ Of these women, 482 remained after exclusion of biopsies which had a worst diagnosis on the newly sectioned slide which was more than 1 grade aside from the consensus diagnosis on the locally produced histology slide. Exclusion of hrHPV‐negative biopsies (87.2% histologically negative biopsies) resulted in a selection of 318 women who met the inclusion criteria.

The definitive diagnoses on the recut biopsy used for new H&E and IHC slides were as follows: 90 no CIN (28.3%), 67 LSIL/CIN1 (21.1%), 95 HSIL/CIN2 (29.9%), 62 HSIL/CIN3 (19.5%), 4 cervical carcinomas (CC) (1.2%). The agreement between the diagnosis on the locally produced slide and the consensus diagnosis on the newly sectioned slide was substantial (Cohen's kappa = 0.747).

### 3.1 Hypermethylation of FAM19A4 and/or miR124‐2 on cytology sample

The methylation positivity for markers FAM19A4 individually and combined with miR124‐2, in the cytology samples of these women according to the definitive histological diagnosis are shown in Table 1. Methylation positivity for markers FAM19A4 and miR124‐2 increased with the severity of the lesion: 23.2% of women with no CIN biopsies were positive for one or more markers compared with 66.1% of women with HSIL/CIN3 biopsies.

**Table 1:** Hypermethylation of single marker FAM19A4, and FAM19A4 and miR124‐2 combined in cytology samples related to different histological grades of CIN (N = 318)

Diagnosis (N)	FAM19A4‐positive (%)	FAM19A4 and/or miR124‐2‐positive (%)
Negative (90)	20 (22.2)	21 (23.2)
LSIL/CIN1 (67)	17 (25.4)	17 (25.4)
HSIL/CIN2 (95)	43 (45.3)	44 (46.3)
HSIL/CIN3 (62)	41 (66.1)	41 (66.1)
CC (4)	4 (100)	4 (100)

FAM19A4 was more frequently positive than miR124‐2 in both ≤LSIL/CIN1 (23.6%, 37/157 compared to 8.9%, 14/157 for miR124‐2, *P* < .001), and in ≥ HSIL/CIN2 (54.7%, 88/161 compared to 37.9%, 61/161 for miR124‐2, *P* < .001).

### 3.2 Expression of p16 and HPV E4 on colposcopic biopsy

The grades of p16 positivity and HPV E4 positivity by worst SIL/CIN grade on the worst histological lesion found on colposcopy‐directed biopsy are shown in Table 2. p16 grade increased with the severity of the lesion, with 85 of 90 (94.4%) no CIN biopsies showing no or patchy p16 staining and all HSIL/CIN3 lesions showing p16 in at least the lower two third of the epithelium or above. None of the no CIN biopsies and only 2 of 64 (3.1%) HSIL/CIN3 and none of the ICC showed any E4 positivity, while 27 of 67 (40.3%) LSIL/CIN1 and 21 of 95 (22.1%) HSIL/CIN2 were E4 positive. The highest percentage of E4 positivity was found among lesions with p16 expression in the lower two third of the epithelium (p16 score 2; 37.3%; 22/59), containing 44.0% (22/50) of all E4‐positive lesions.

**Table 2:** p16 and E4 expression in different grades of CIN (n = 318) on worst histological lesion on colposcopy‐directed biopsy

p16	Diagnosis				
	No CIN (N = 90)	LSIL/CIN1 (N = 67)	HSIL/CIN2 (N = 95)	HSIL/CIN3 (N = 62)	CC (N = 4)
	E4 positive	E4 positive	E4 positive	E4 positive	E4 positive
0 ‐ negative (N = 103)	0/85 (0%)	7/15 (47%)	0/3 (0%)	0/0 (0%)	0/0 (0%)
1 ‐ lower 1/3 (N = 27)	0/2 (0%)	7/20 (35%)	1/5 (20%)	0/0 (0%)	0/0 (0%)
2 ‐ lower 2/3 (N = 59)	0/3 (0%)	7/15 (47%)	13/27 (48%)	2/14 (14%)	0/0 (0%)
3 ‐>lower 2/3 (N = 129)	0/0 (0%)	6/17 (35%)	7/60 (12%)	0/48 (0%)	0/4 (0%)

### 3.3 Relation between p16 and E4, and p16 and methylation

**Figure 1:**
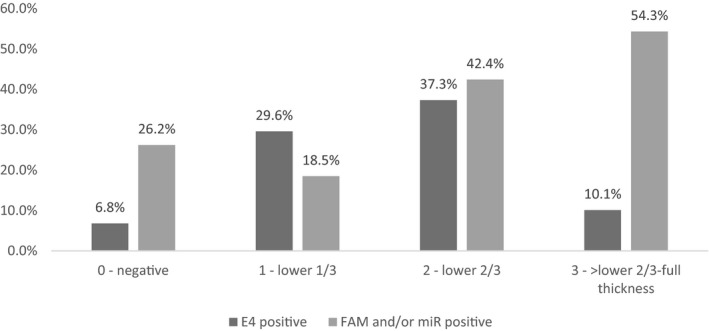
E4 positivity on biopsy (Negative‐ICC) and methylation positivity on cytology sample (FAM19A4 and/or miR124‐2) in women with lesions expressing different grades of p16

Figure 1 shows the relation between p16 grade of worst lesion on colposcopy‐directed biopsy, methylation of markers FAM19A4 and miR124‐2 as detected on cytology sample and E4 grade of worst lesion on biopsy.

Expression of E4 increased with increasing p16 expression with the highest percentage of E4 positivity seen in lesions with p16 in the lower two third of the epithelium (37.3%). However, in lesions that express p16 above the lower two third of the epithelium, E4 positivity dropped to 10.1% (*P* < .001). In biopsies showing p16 up to two third of the epithelium (n = 189), there was a significant correlation between p16 expression and E4 expression (r = 0.378, *P* < .001).

Of the women with lesions showing no or patchy p16 expression, 26.2% (27/103) had a FAM19A4 and/or miR124‐2 methylation‐positive cytology sample. The frequency dropped to 18.5% methylation‐positive cytology samples in women with lesions showing p16 in the lower third of the epithelium but increased to 42.4% methylation positivity in women with p16 in the lower two third of the lesion and 54.3% in women with lesions showing p16 above the lower two third of the epithelium. There was a significant correlation between grade of p16 expression on biopsy and methylation positivity on cytology sample (r = 0.261, *P* < .001).

### 3.4 Relation between methylation and p16/E4 expression in ≥LSIL/CIN1 lesions

**Figure 2:**
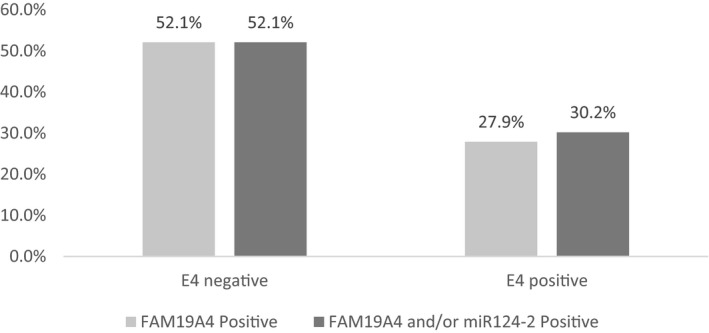
Positivity for marker FAM19A4 and markers FAM19A4 and miR124‐2 combined (and/or) in cytology samples, in E4 negative and E4 positive LSIL/CIN1 lesions with diffuse p16 expression in at least the lower 1/3 of the epithelium (p16 ≥ 1)

The relation between hypermethylation of markers FAM19A4 and/or miR124‐2 on cytology sampling and E4/p16 on the worst histological lesion on biopsy was explored by selecting all LSIL/CIN1‐3 lesions and ICCs with diffuse p16 expression in at least the lower third of the epithelium (n = 210). In this group, an inverse relation was found between E4 and FAM19A4/miR124‐2. Figure 2 shows the positivity for marker FAM19A4 as a single marker and combined with (and/or) miR124‐2 in E4‐negative and E4‐positive lesions. Of the biopsies negative for HPV E4, 52.1% belonged to women with cytology samples positive for methylation marker FAM19A4, which was a significantly higher percentage than the 27.9% of women with E4‐positive biopsies who had a cytology sample positive for methylation marker FAM19A4 (*P* = .005). The same pattern was observed for the combination of FAM19A4 and/or miR124‐2 methylation markers, which was positive in 52.1% of the women with E4‐negative biopsies and 30.2% of the women with E4‐positive biopsies (*P* = .010).

### 3.5 Relation of p16/E4 status of worst colposcopic biopsy lesion and methylation status to detection of ≥HSIL/CIN2 on LEEP treatment

To find out the relation between p16/E4 marker patterns on colposcopy‐directed biopsy or FAM/miR on the cytology sample and a ≥HSIL/CIN2 diagnosis on LEEP, 119 women with HSIL/CIN2‐3 on biopsy treated by LEEP were investigated. Of these women, 22 (18.5%) with a mean age of 40.1y had a worst diagnosis of ≤LSIL/CIN1 on LEEP, and 97 (81.5%) women with a mean age of 37.5y had a worst diagnosis of ≥HSIL/CIN2 on LEEP.

Table 3 shows the relation between biopsy diagnosis, worst diagnosis on LEEP specimen, and p16/E4 on biopsy and methylation positivity on cytology sample. Of the treated ≥HSIL/CIN2 lesions, 17 (14.3%) were E4 positive and 102 (85.7%) were E4 negative. However, grade of p16 expression in relation to E4 expression was important. Lesions with p16 expression restricted to the lower third of the epithelium, or less, and all lesions with E4 positivity were classified as combined IHC negative. All biopsies expressing p16 in at least the lower two third of the epithelium and no E4 were considered combined IHC positive. Of the combined IHC‐negative lesions on biopsy, 65.2% (15/23) were confirmed as ≥HSIL/CIN2 lesions on LEEP, and 85.4% (82/96) combined IHC‐positive lesions on biopsy were ≥HSIL/CIN2 on LEEP (*P* = .025). The same was done for the outcome of ≥HSIL/CIN3 on LEEP, showing that 26.1% (6/23) of women with combined IHC‐negative biopsies and 49.0% (47/96) of women with combined IHC‐positive biopsies had ≥HSIL/CIN3 on LEEP (*P* = .047).

**Table 3:** Women treated after HSIL/CIN2‐3 on biopsy (N = 119) and the relation to p16/E4 positivity on biopsy, methylation positivity (FAM19A4 and/or miR124‐2) on cytology sample and worst diagnosis on LEEP

Worst diagnosis of CIN2/3 on biopsy (n)		Worst diagnosis LEEP		*P*‐value	Worst diagnosis LEEP		*P*‐value
		≤LSIL/CIN1	≥HSIL/CIN2		≤HSIL/CIN2	≥HSIL/CIN3	
		Count (%)	Count (%)		Count (%)	Count (%)	
Combined IHC on biopsy	Combined IHC negative (23)	8 (34.8)	15 (65.2)	.025	17 (73.9)	6 (26.1)	.047
	Combined IHC positive (96)	14 (14.6)	82 (85.4)		49 (51.0)	47 (49.0)	
Methylation on cytology	Methylation negative (47)	11 (23.4)	36 (76.6)	.264	31 (66.0)	16 (34.0)	.063
	Methylation positive (72)	11 (15.3)	61 (84.7)		35 (48.6)	37 (51.4)	

Note

Lesions with p16 expression restricted to the lower third of the epithelium, or less, and all lesions with E4 positivity regardless of p16 expression were classified as combined IHC negative. All biopsies expressing p16 in at least the lower two third of the epithelium and no E4 were considered combined IHC positive.

Of the 119 women who were treated after a ≥HSIL/CIN2 lesion on biopsy, 60.5% (72/119) had a FAM and/or miR methylation‐positive sample and 39.5% (47/119) had a methylation‐negative sample. ≥HSIL/CIN2 on LEEP occurred in 84.7% (61/72) methylation‐positive women and 76.6% (36/47) methylation‐negative women (*P* = .264). Among the methylation‐negative women there were 34.0% (16/47) with ≥HSIL/CIN3 on LEEP (all HSIL/CIN3), and among women with methylation‐positive samples there were 51.4% (37/72) with ≥HSIL/CIN3 (including two CCs; *P* = .063).

## 4 DISCUSSION

This study shows that in women who have hrHPV‐positive SIL/CIN there are complex relationships between expression patterns of the widely used immunohistochemical marker p16 and novel immunohistochemical marker E4 on biopsy. They also show relationships between these biopsy markers and the methylation markers FAM19A4/miR124‐2 on cervical cytology sample. Firstly, there is an inverse relation between productive infection as shown by HPV E4 positivity in the worst lesion present on biopsy, and methylation of markers FAM19A4 and miR124‐2 on cytology which are associated with transforming HPV infection. In women with diffusely p16 positive ≥LSIL/CIN1, a difference in FAM19A4 and/or miR124‐2 positivity rates was found between women with lesions expressing E4 and those who did not: 52.1% of women with E4‐negative and 30.2% with E4‐positive biopsies were positive for the methylation markers (*P* = .010).

These results are consistent with previous studies on selective series of expression of somatic tumor suppressor gene methylation markers and IHC for p16 and E4 and extend the observations to women attending routine colposcopy for an abnormal cervical cytology sample.^17^ Van Zummeren et al found an inverse relation between HPV E4 expression and methylation positivity for marker combination CADM1/MAL/miR124‐2 detected on biopsy.

In addition, previous work showed that a cumulative immunoscore of p16 and Ki‐67 improved accuracy and reproducibility of CIN grading compared to current practice.^18^ These studies and the present study showed that whereas CC shows no E4 expression and often expresses both p16 and methylation marker positivity, LSIL/CIN1 and HSIL/CIN2 are very heterogeneous and complex groups, consisting of productive infections expressing E4 and transforming lesions expressing variable p16, some of which show methylation marker positivity. The use of the E4 immunomarker and methylation markers in this group could offer more detailed information beyond current SIL/CIN grading practice and the use of p16 alone.

E4 positivity was related to p16 positivity and histology in hrHPV‐positive biopsies in a rather complex way. E4 was absent in no CIN biopsies, was 40% in LSIL/CIN1, decreased to 22% in HSIL/CIN2 and 3% in HSIL/CIN3, and was again absent in cancer. The rate of E4 positivity increased with grade of p16 expression (r = 0.378) up to 37% in lesions with p16 in the lower two third of the epithelium, but fell to 10% when p16 expression was above two third of the epithelium.

Hypermethylation of FAM19A4 and/or miR124‐2 on cervical cytology sample increased with the grade of SIL/CIN, but there was some positivity (24%) in ≤LSIL/CIN1, rising to 66% in HSIL/CIN3 and was seen in all cancers. Methylation positivity progressively increased with grade of diffuse p16 positivity (18.5% in lesions with p16 in the lower third to 54.3% in lesions with p16 up to full thickness). There was also some (26.2%) methylation in samples of women with lesions negative or patchy for p16 IHC.

p16 is widely used as marker of transformation in hrHPV‐associated CC and precancer, but this study shows that it is also expressed in up to the lower two third of cervical epithelium in lesions showing completion of the HPV life cycle and HPV production by E4 expression, and that increasing extent of p16 expression up to two third of the epithelium is associated with more frequent E4 expression. This is consistent with the expression of p16 as a surrogate for hrHPV E7 expression and the important role of E7 in driving the increased epithelial proliferation above the basal layer necessary for viral reproduction, as well as playing a part in driving neoplastic transformation. This finding explains the difficulty of using p16 on its own as a reliable transformation marker in SIL/CIN diagnosis. Only expression of p16 above two third of the epithelium is associated with infrequent E4 expression showing loss of viral life cycle completion with virus production and increased frequency of methylation of tumor suppressor genes.

We also found evidence that p16 expression and E4 positivity on the worst colposcopy‐directed biopsy related to the frequency of finding HSIL/CIN2 and HSIL/CIN3 on LEEP in women treated for ≥HSIL/CIN2. Our results support the view that lesions with diffuse p16 expression limited to the basal third and E4 positivity are likely to be smaller and/or more regressive lesions. Luttmer et al have previously shown an increasing positivity rate for FAM19A4 analysis performed on cervicovaginal self‐samples and cervical cytology samples with the severity and volume of the lesion.^19^ In the present study, a methylation‐positive cytology sample showed no significant relation to treatment outcome.

This study was performed in a selected group of women who were referred for colposcopic examination after an abnormal Pap‐smear result, who had a hrHPV‐positive cervical biopsy and had cervical cytology samples suitable for methylation testing. Methylation status was only determined in the cervical cytology samples and not on biopsy. However, a previous study from van Baars et al confirmed that the methylation status of the cervical cytology sample represents the methylation status of the worst lesion present on the cervix when taking biopsies of all lesions detected during colposcopy together with a random biopsy of normal appearing tissue.^9^ Sensitivity of colposcopy for the detection of ≥HSIL/CIN2 is between 50% and 70%^20^, ^21^, ^22^, ^23^ and methylation positivity on cytology in the absence of high‐grade disease on biopsy could possibly originate from a high‐grade lesion that was missed on colposcopy.

We used a reference standard diagnosis in which the opinion of at least three pathologists was reflected, and which assured that the worst lesion was present in all tested material. It is known that reproducibility of diagnosis of LSIL/CIN1 and HSIL/CIN2 is limited and intra‐ and interobserver agreement is moderate.^5^, ^24^ By excluding women in which the SIL/CIN colposcopy‐directed biopsy diagnosis of the worst lesion on the newly produced slide differed more than one histological grade from the consensus diagnosis on the local slide, we attempted to use the most accurate reference standard diagnosis as possible, still allowing for biomarker expression identification.

Large prospective studies are needed to investigate the outcome of E4‐positive and negative LSIL/CIN1 and HSIL/CIN2. Currently available methylation markers have shown good clinical performance in the detection of advanced transforming precursor lesions and CC in hrHPV‐positive women. In addition, results from a large follow‐up study have shown a low CC risk for hrHPV‐positive women with a negative FAM19A4/miR124‐2 triage test result, offering an objective triage test in hrHPV‐based screening programs.^25^ We found that FAM19A4 has the major contribution to predicting the presence of HSIL/CIN3 as compared to miR124‐2. We scored methylation markers positive or negative, without taking into account the hypermethylation levels. Studies on tumor suppressor genes CADM1 and MAL showed increasing levels of hypermethylation toward CC.^11^ Future studies are needed to demonstrate whether FAM19A4 and miR124‐2 methylation positivity levels differ between women with E4‐positive and E4‐negative lesions.

Our results show that there is a possibility of more specific diagnosis of CIN when using grading of patterns of immunohistochemical expression of HPV E4 in combination with p16^INK4A^. Lesions with diffuse, but limited p16 positivity and E4 positivity most likely represent early transforming and productive lesions with variable HPV life cycle completion and might have a higher probability of spontaneous regression. Extensively p16‐positive, E4‐negative lesions in patients, and those with a methylation positive cervical cytology sample are more often ≥HSIL/CIN3 lesions and most likely represent advanced transforming lesions that require LEEP treatment. Further studies are required to establish the progression risk of different biomarker expression patterns in women not treated by LEEP.

## Data Availability

The authors confirm that the data supporting the findings of this study are available within the article.
